# Effects of exogenous plant regulators on growth and development of “Kyoho” grape under salt alkali stress

**DOI:** 10.3389/fpls.2023.1274684

**Published:** 2023-12-14

**Authors:** Maoxiang Zhao, Jiajia Li, Xiangneng Shi, M. Sanaullah Malik, Yi Quan, Dinghan Guo, Lei Wang, Shiping Wang

**Affiliations:** ^1^ Department of Plant Science, School of Agriculture and Biology, Shanghai Jiao Tong University, Shanghai, China; ^2^ Graduate School of Environmental and Life Science, Okayama University, Okayama, Japan; ^3^ Sinochem Agriculture Holdings, Beijing, China

**Keywords:** salt alkali stress, γ-aminobutyric acid, salicylic acid, brassinolide, grapevine growth, fruit quality

## Abstract

Salinity is one of the major abiotic stresses besides drought and cold stress. The application of plant growth regulators (PGRs) is an effective method to mitigate yield losses caused by salinity. However, we investigated the effects of exogenous regulatory substances (γ-aminobutyric acid (GABA), salicylic acid (SA), and brassinolide (BR) on the growth and development of “Kyoho” grapevine under salt stress. The results showed that exogenous regulators GABA, SA, and BR alleviated the inhibition of grape growth by saline stress and regulated the effects of salinity stress on grape fruit development and quality. All three regulators significantly increased fruit set, cross-sectional diameter, weight per unit, and anthocyanin content. In conclusion, this study provides a theoretical basis for grape production practices by using exogenous aminobutyric acid (GABA), salicylic acid (SA), and brassinolide (BR) to mitigate the hazards of salinity stress.

## 1 Introduction

Grapevine has become one of the most widely cultivated horticultural crops in China due to its adaptability and economic efficiency. The grapevine industry has also significantly improved agricultural productivity and revenue for farmers across various regions (Deng et al., 2021). China is one of the leading countries producing grapes. In the last few years, grape production in China has observed an overall upward trend. The third-largest saline area of land in the world is found in China. Numerous grape orchards have been planted on saline land to reduce the area of cultivable land occupied by vineyards needed for food production. However, the high levels of salinity in the soil have a negative impact on the growth, yield, and quality of the grapes. To reduce the amount of arable land occupied by vineyards for growing food, various vineyards have been established on saline land, and the highly saline soils seriously affect the growth, yield, and quality of grapes (Mirás-Avalos and Intrigliolo, 2017; Wei et al., 2023).

Soils with high pH or salt concentration are referred to as saline soils. Additionally, they have a significant impact on crop yield and quality. Numerous studies have shown that salt alkali stress inhibits plant growth and development. Seedling height, above-ground bioaccumulation, and root bioaccumulation were significantly lower in small rye (*Secale cereale* L.) under saline conditions than in the control treatment (Mohammadi Alagoz et al., 2023). In addition, similar results were observed in melon, tomato, and mung bean plants (Torabian et al., 2018; Pérez-Labrada et al., 2019; Sarabi et al., 2019). Furthermore, different salinity concentrations also have a different effect on several plants. The saline solution at a concentration of 25 mmol L^-1^ increased the fresh weight of the roots of the tomato seedlings; when the saline solution concentration increased to 75 mmol L^-1^, the fresh weight of seedlings roots was significantly lower than that of the control (Alves et al., 2018). Similarly, the plant height of Rubus crataegifolius seedlings subjected to low alkaline salt concentration was higher than the control plants (Li et al., 2016). The plant growth was inhibited to different degrees with increasing stress concentration. In terms of phenology, plants such as sea buckthorn (Qin et al., 2009), sweet pepper (Giorio et al., 2020), and mulberry (Zhang et al., 2022) all showed yellowing of old basal leaves, water loss, and even abscission after salt alkali stress. Therefore, it is of great importance to explore the application of exogenous plant regulators to enhance the ability of plants to cope with salt alkali stress during growth and development.

γ-aminobutyric acid (GABA) is a natural non-protein amino acid that is widely found in plants and animals (Sarasa et al., 2020). When plants are subjected to abiotic stresses such as salt, alkali, drought, and high temperatures, it leads to a rapid accumulation of GABA in plants, which enhances their resistance to unfavorable stresses (Mishra et al., 2023). Salicylic acid (SA) and brassinolide (BR) are endogenous plant hormones that are involved in regulating various physiological activities. When plants face various stresses, they release plenty of endogenous SA and BR to alleviate the damage caused by these stresses (Guo et al., 2018). In recent years, there have also been many new developments in research on the relationship between SA and plant salt alkali stress. On the other hand, SA regulates plant growth, development, and photosynthetic phenomenon (Jangra et al., 2022). SA stimulated the activity of antioxidant enzymes in plants. In response to salt alkali stress, SA mitigated the damage by increasing the levels of chlorophyll, glycine betaine, proline, total phenol, numerous antioxidants, and defense-related enzyme activities (Alam et al., 2022). The adverse effects of salt alkali stress on *Xanthium sibiricum* Patr. are reduced via exogenous BR (Zhou et al., 2023). Similar research revealed that exogenous BR sprayed on cucumber leaves during salt alkali stress reduced the concentrations of ROS, MDA, and electrical conductivity in plant leaves while dramatically enhancing the activity of many antioxidant enzymes. In plants, the content of redox substances is strictly regulated. In addition, it has been reported that the rate of photosynthetic activity has been significantly improved (He et al., 2022). In terms of fruit quality improvement, BR spraying at appropriate mass concentrations was able to increase the yield of sugar beet tubers but had no significant effect on total sugar content (Lv et al., 2019).

In this study, the effects of salt alkali stress on photosynthetic physiology, antioxidant properties and fruit quality of grapevine were investigated. Moreover, GABA, SA, and BR have mitigating effects against salinity damage in grapes as well, and the extent of mitigation was preliminarily explored. We found that most of the parameters associated with photosynthetic physiology, antioxidants, and fruit quality were increased under exogenous GABA, SA, and BR treatments. These findings laid a solid foundation for improving grape photosynthetic properties and fruit quality by using exogenous plant regulators in grapevine cultivation.

## 2 Materials and methods

### 2.1 Experimental materials and design

The experiment was carried out by planting four-year-old “Kyoho” grape cuttings (Bred in our laboratory) in a rain-proof glass greenhouse at the Agricultural Engineering Training Center of Shanghai Jiao Tong University, Shanghai (121.45°E, 31.04°N). With a light-permeable roof over the experimental material, the root restriction was used as a cultivation method. The substrate was made of soil, organic matter, and perlite at a ratio of 4:1:1. The plant spacing and the row spacing were both 40 cm. Seven biological replicates were used for each treatment. An automatic irrigation system was carried out using an integrated water and fertilizer unit.

A total of 35 healthy and uniformly growing grapevines were selected and marked before the trial. Five treatments were set up in this trial, Control: water sprayed under normal soil conditions; S: four types of salts, NaCl, Na_2_SO_4_, NaHCO_3_, and Na_2_CO_3_, are mixed in a ratio of 1:9:9:1 to create saline soil conditions, water supplement was same as the control group; S+GABA: 100 mmol L^-1^ aminobutyric acid (GABA) solution under saline soil conditions; S+SA: 0.1 mg L^-1^ salicylic acid (SA) solution under saline soil conditions; S+BR: 0.1 mg L^-1^ brassinolide (BR) solution under saline soil conditions; S+SA: 0.1 mg L^-1^ salicylic acid (SA) solution in saline soils; S+BR: 0.1 mg L^-1^ brassinolide (BR) solution in saline soils. In this study, the critical phenological periods were: May 2 (flowering); May 9 (0 day (D) after saline stress, end of flowering); May 9 (0 (D) after saline stress, grape fruit expansion) to July 11 (63 (D) after saline stress, grape fruit expansion); and July 11 (63 (D) after saline stress, grape fruit expansion); July 11 (at 63 (D) after saline stress, verasion stage); and August 1 (at 84 (D) after saline stress, ripening stage).

### 2.2 Determination of physical and chemical properties of soil

After each salinity stress treatment, the samples were taken by diagonal method using a soil extractor, and the samples taken for each treatment were mixed thoroughly. The samples were spread evenly on an iron tray and then baked in an oven at 90 °C for 2 d. After that, the samples were ground using a pulverizer and stored in a dry and cool place. Subsequently, a 50 mL test tube was prepared, 40 mL of de-carbonated distilled water was added, and then 8 g (sample mass: solvent mass = 15) of sieved soil samples were added. The soil samples were stirred thoroughly with a glass rod for 2 min to dissolve fully, then centrifuged at 6000 rpm for 5 min at room temperature, then the supernatant was taken. Soil pH values were determined with a pH meter. Soil salt content was determined by the mass difference method (Kennedy et al., 2001). After aspirating 20 mL of supernatant 50 mL beaker then dried at 180 °C, during which 2-4 drops of hydrogen peroxide were added. It was then weighed on a one-in-ten thousand balance, and each treatment was repeated with three biological replicates.

### 2.3 Measurement of new shoot diameter and new shoot length

At 0 D of salinity stress, six vines were randomly selected and marked with a black marker. The diameter of the marked shoot base was measured at D 0, 21, 35, 49, 63, 77, 91, and 105 using vernier calipers as well as the new shoot length was measured. Each treatment was repeated in three biological replicates.

### 2.4 Measurement of leaf physiological indicators

At 0 D, 21 D, 35 D, 49 D, 63 D, and 84 D of salinity stress, the chlorophyll content of the 15 leaves was measured using a hand-held SPAD-502 chlorophyll meter. At 112 D after salinity stress, six plants were selected for each treatment, and the number of all existing leaves on their main stem and lateral branches was counted. The sum of the number of traces at leaf abscission and the existing number of leaves was recorded as the total number of leaves.

Measurements were recorded at 11:00-11:30 am on D 0, D 21, D 35, D49, D 63 D, and D 84 of the salinity treatment. A plant efficiency meter (model Handy PEA+) provided by Hansatech was prepared, and healthy plant leaves were selected. After avoiding the main leaf veins, the leaves were placed in a dark acclimation folder for about half an hour to avoid light acclimation. This was followed by saturated pulsed light irradiation with an intensity of 5000 mol m^-2^ s^-1^ for 0.8 s. The relevant parameters were determined. All treatment was repeated in three biological replicates.

### 2.5 Measurement of leaf photosynthetic parameters

The leaves were measured at 0 D, 21 D, 35 D, 49 D, 63 D and 84 D after the saline treatment, using a CIRAS-3 photosynthesis meter from PP Systems and a constant intensity of 1300 μmol mol^-1^ of artificial light source, measured between 8:00 a.m. and 9:30 a.m. The response curves were measured between 9:30 a.m. and 11:30 a.m. The light response curves were then analyzed with a foliar drift model with a gradient of 0, 50, 100, 200, 500, 800, 1000, 1500, 1800, and 2000 μmol mol^-1^ of light quantum flux density (PPFD). All treatment was repeated in three biological replicates.

### 2.6 Determination of relative conductivity

The relative conductivity of grape leaves was measured using the immersion method. Grapevine plants were selected at 0 D, 21 D, 35 D, 49 D, 63 D, and 84 D after saline treatment was applied through the bottom of the leaves at 6-9 nodes washed with deionized water. Holes were created on the leaves’ surface using a puncher, then placed in 50 mL test tubes, and 15 mL of deionized water was added. After 24 h of soaking at room temperature, the conductivity values were determined before and after boiling by a conductivity meter. The conductivity values of the extracts before and after boiling were measured and calculated. Each treatment was replicated three times.

### 2.7 Determination of relative water content

At 0, 21, 35, 49, 63 and 84 Days of the salinity stress treatment, leaves were taken from grapevine plants at the 6-9 node leaf position counting from the base and placed in ice to be brought back to the laboratory rapidly. Leaves were first rinsed with tap water, then cleaned with deionized water properly, followed by drying with paper towels. After that fresh weight of the leaves was measured by removing the petiole and then the samples were immersed in deionized water for 3 hours. After that, the extra water was removed from the surface with absorbent paper, and weighed immediately, that is the turgid weight. Then dried at 70 °C to a constant weight, followed by cooling at a constant temperature. Finally, dry weight was measured. All treatment was repeated in three biological replicates.

### 2.8 Determination of physiological indicators and antioxidant enzyme activity

The soluble titrant acid is measured by a potentiometric titrator (ZD-3A, SHN), and the soluble solid content is measured by a handheld sugar meter (ATAGO, JPN). Fruit hardness is measured by a hardness tester (LD-GY-4, CHN).

Firstly, 0.250 g of the leaf tissue with veins was removed on a one-thousand balance by putting it into a 2 mL test tube pre-chilled with liquid nitrogen. After that, 2-3 mL of phosphate buffer (0.05 mol L^-1^, PH=7.8) was added and then the samples were ground, followed by centrifugation. The volume of the samples was kept fixed to 9 mL using phosphate buffer. The enzymes were centrifuged at 4 °C for 5 min in a high-speed centrifuge at 6000 rpm (need to be pre-cooled at 4 °C in advance), and then the enzyme solution was removed and placed in an ice box. The indicators were analyzed on the same day, with three replicates for each treatment. The SOD, POD and CAT enzyme activities were measured according to the previously optimized method in the laboratory. All treatment was repeated in three biological replicates.

### 2.9 Determination of anthocyanin

The total anthocyanin content in the pericarp was determined by the pH differential method. First, the pericarp was ground to a powder form using a mortar pre-cooled with liquid nitrogen. Weighed 1 g of powder into a centrifuge tube and then 10 mL of formic acid-methanol solution (1%) was added. After that it was kept overnight at 4°C on a low-temperature shaking bed with shading. The pericarp was white when the extraction was completed. Then, centrifuged at 4°C for 10 min at 8000 rpm. 1.0 mL of the extract was added in separate tubes; the solution volume of one tube was fixed to 10 mL with buffer (pH=1.0) and the other was fixed to 10 mL with buffer pH=4.5. The absorbance at 520 nm and 700 nm in the UV spectrophotometry was measured after equilibration for two hours. For each treatment, three biological replicates were set up.

### 2.10 Data analysis

Experimental data was recorded and icons were plotted using Microsoft Excel for Mac software. Duncan’s technique (P<0.05) was applied for significant difference analysis after the data were processed using SPSS 26.0 software for statistical analysis.

## 3 Results

### 3.1 Effect of salinity stress on soil physicochemical properties

**Figure 1:**
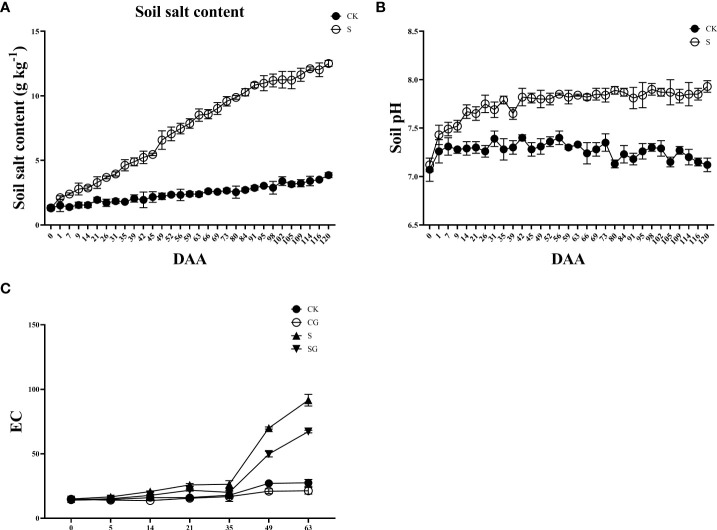
Salinity stress on soil physicochemical properties. **(A)** Soil salt content. **(B)** Soil pH. **(C)** Soil pH.

The saline group’s pre-experiment soil salt content was 1.32 g kg^-1^, which was not substantially different from the control group as shown in **Figure 1A**. The soil’s salt concentration rose to 12.51 g kg^-1^ when salt alkali stress intensified. The trend in soil pH was similar at the final stage of the experiment (**Figure 1B**). The soils in the control and saline groups were normal before the start of the experiment with pH values of 7.07 and 7.12, for each group, respectively. With the degree of salt alkali stress deepening, the pH value of the saline soil reached 7.5 at DAA 59 (day after anthesis), which became a typical alkaline soil. At the end of the experiment (DAA 120), the pH of the saline soil group was 7.93, which was 0.81 units higher than that of the control group. In addition, the trend in soil EC value showed a slow change at first and a sharp increase in the later stage of fruit ripening, and the saline group was significantly higher than the other treatment groups (**Figure 1C**).

### 3.2 Effects of exogenous regulators on phenotypic and physicochemical indexes of grape leaves under saline stress

**Figure 2:**
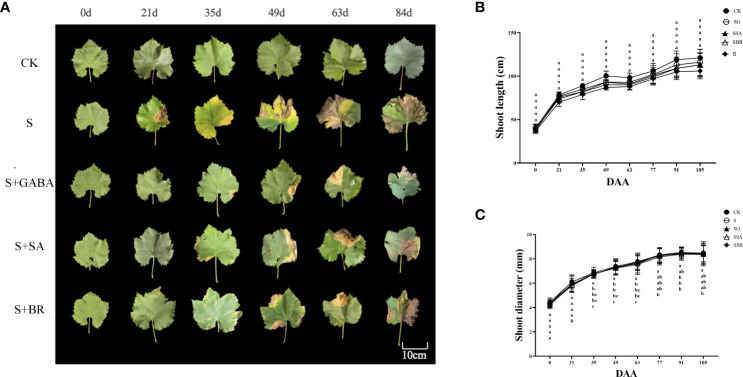
Effects of plant regulators on the leaf phenotype of grapevines under saline-alkali stress. **(A)** Leaf phenotype. **(B)** Shoot length. **(C)** Shoot diameter. Significant differences among samples have been displayed by a-e letters (one-way ANOVA, P<0.05). Error bars = ± SE (n≥3).

Before the start of the experiment, the leaf phenotype showed a healthy state in all groups. Yellowing and scorching began to appear in the old leaves of group S from the 21 D of salinity stress as shown in **Figure 2A**. After the exogenous treatment of regulators GABA, SA and BR, there were no significant changes in the grape leaves as compared to the control group. Overall, when compared with BR, the effects of GABA and SA were more persistent. The diameter of new shoots in all treatment groups showed a sharp increase followed by a slow increase as shown in **Figure 2B**. At 105 D after salinity stress, the diameter of new shoots in each group was twice as large as the initial value. The new tip length of each group showed a sharp increase followed by a slight decrease and then a slow increase, as shown in **Figure 2C**. Moreover, the S group was always lower than the control group. By the 105 D of saline stress, the new shoot length of the S group was only 87.75% of that of the control group. However, from 77 D salinity stress, there was no significant difference between the S group and the S+GABA, S+SA, and S+BR groups.

### 3.3 Effect of exogenous regulators on photosynthetic properties of grape leaves under salinity stress

**Figure 3:**
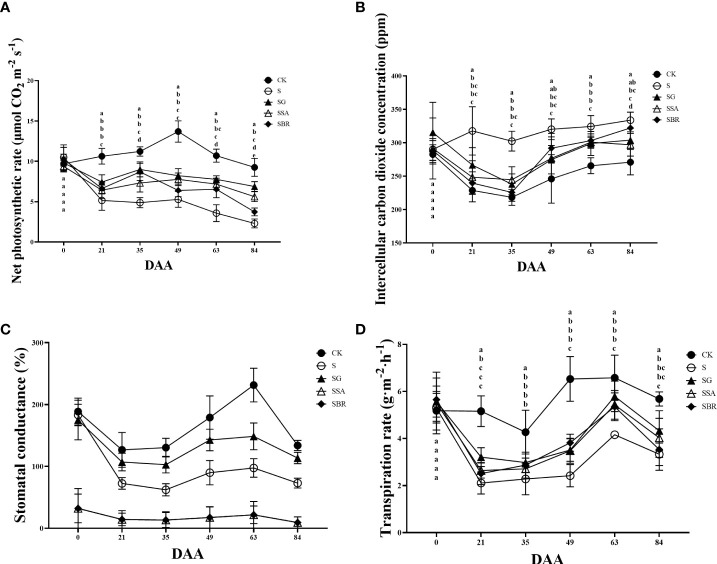
Effects of plant regulators on the net photosynthetic rate of grapevine leaves under saline-alkali stress. **(A)** Net photosynthetic rate. **(B)** Intercellular CO_2_ concentration. **(C)** Stomatal conductance. **(D)** Transpiration rate. Significant differences among samples have been displayed by a-e letters (one-way ANOVA, P<0.05). Error bars = ± SE (n≥3).

The net photosynthetic rates between CK and four treatment groups was non-significant under salinity before the experiment (**Figure 3A**). Under normal conditions, the net photosynthetic rate showed an increasing pattern and start declining while all groups under saline stress showed a decreasing trend than the control group. However, the net photosynthetic rate increased to some extent after spraying the exogenous regulators, comparatively, the net photosynthetic rate of the S+GABA group was the closest to that of the control group. It indicated that the spraying of the exogenous regulator GABA was the most effective in alleviating saline stress. There was no significant difference in the intercellular CO_2_ concentration between the control and S, S+SA, and S+BR groups before the treatment, as shown in **Figure 3B**. The intercellular CO_2_ concentration in all treatment groups showed a decreasing and then increasing trend within three months after the salinity stress treatment. The intercellular CO_2_ concentration in the S+BR group began to increase than that in the S+SA and S+GABA groups at 49 D post-stress and was not significantly different from that in the S-treated group at 84 D post-stress.

The stomatal conductance of the four different treatment groups under saline stress was not significantly different from that of the control group as shown in **Figure 3C**. All groups of grape plants’ stomatal conductance exhibited a pattern of reducing, then increasing, and subsequently decreasing as the plants grew. Additionally, the overall effect of all three regulators was reduced by the inhibition of stomatal conductance under saline stress.

Throughout the growth period, the transpiration rate of grapevine plants under control conditions or saline stress exhibited a trend of declining, then rising followed by decreasing as illustrated in **Figure 3D**. The transpiration rate under control conditions was always higher than that of the groups under saline stress. Among the three exogenous regulators, only GABA still promoted transpiration, which increased by 21.02% compared to the S treatment group.

### 3.4 Effect of exogenous regulators on the light response curve of grape leaves under salinity stress

**Figure 4:**
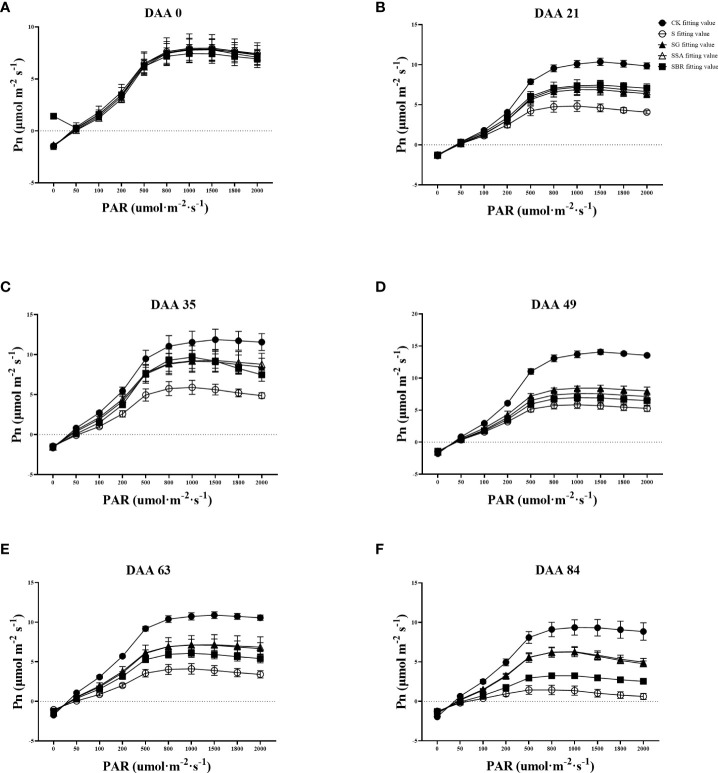
Effects of plant regulators on light response curves of grapevine leaves under salt alkali stress. **(A-F)**, respectively, represents the light response curves of saline-alkali stress treatment on days 0^th^, 21^st^, 35^th^, 49^th^, 63^rd,^ and 84^rd^).

The CK control group’s light absorption values gradually rose as illustrated in **Figures 4A–F** and **Table 1**. In contrast, saline stress caused the light penetration level for each treatment to occur significantly earlier. In addition, the three treatment groups (GABA, SA and BR) increased from the initial values of 1272.497 μmol m^-2^ s^-1^, 1227.037 μmol m^-2^ s^-1^ and 1210.380 μmol m^-2^ s^-1^ to 23.61%, 24.05% and 25.30% of the initial values, respectively.

Table 1Parameters of light response curves.

**Table:** 

Days after saline-alkali stress	Treatment	AQE	Pn max	LSP	LCP	R_d_	R^2^ of Model fitting
		(μmol·μmol^-1^)	(μmol·m^-2^·s^-1^)	(μmol·m^-2^·s^-1^)	>(μmol·m^-2^·s^-1^)	(μmol·m^-2^·s^-1^)	
0	CK	0.036 ± 0.008 a	7.983 ± 0.146 a	1240.473 ± 67.355 a	46.218 ± 3.529 a	1.472 ± 0.199 a	0.991 ± 0.007 a
	S	0.038 ± 0.007 a	8.068 ± 1.360 a	1274.127 ± 93.815 a	43.377 ± 9.334 a	1.452 ± 0.052 a	0.993 ± 0.004 a
	S+GABA	0.045 ± 0.012 a	7.883 ± 1.114 a	1272.497 ± 131.749 a	39.860 ± 9.907 a	1.513 ± 0.039 a	0.990 ± 0.003 a
	S+SA	0.032 ± 0.003 a	7.921 ± 1.034 a	1227.037 ± 24.855 a	49.640 ± 3.570 a	1.438 ± 0.100 a	0.990 ± 0.005 a
	S+BR	0.039 ± 0.002 a	7.507 ± 0.907 a	1210.380 ± 33.147 a	40.744 ± 2.857 a	1.424 ± 0.114 a	0.986 ± 0.005 a
21	CK	0.038 ± 0.001 a	10.387 ± 0.462 a	1373.303 ± 27.810 a	39.143 ± 2.895 a	1.388 ± 0.076 a	0.990 ± 0.008 a
	S	0.033 ± 0.005 a	4.841 ± 0.660 c	1037.613 ± 84.094 b	45.423 ± 6.861 a	1.272 ± 0.045 a	0.992 ± 0.002 a
	S+GABA	0.033 ± 0.003 a	6.971 ± 0.729 b	1236.003 ± 67.147 a	41.241 ± 4.338 a	1.235 ± 0.198 a	0.992 ± 0.001 a
	S+SA	0.032 ± 0.004 a	7.294 ± 0.902 b	1236.143 ± 64.480 a	44.647 ± 6.778 a	1.282 ± 0.179 a	0.990 ± 0.004 a
	S+BR	0.037 ± 0.006 a	7.506 ± 0.460 b	1286.613 ± 114.031 a	39.104 ± 8.627 a	1.270 ± 0.166 a	0.976 ± 0.014 a
35	CK	0.057 ± 0.001 a	11.884 ± 1.332 a	1470.243 ± 123.204 a	32.100 ± 1.585 c	1.680 ± 0.050 a	0.991 ± 0.003 a
	S	0.031 ± 0.003 c	5.915 ± 0.852 c	1079.570 ± 81.683 b	53.651 ± 3.656 a	1.455 ± 0.087 b	0.986 ± 0.004 a
	S+GABA	0.044 ± 0.005 b	9.249 ± 1.360 b	1220.183 ± 69.978 b	39.471 ± 3.866 bc	1.564 ± 0.068 ab	0.985 ± 0.007 a
	S+SA	0.048 ± 0.004 b	9.348 ± 1.350 b	1271.763 ± 95.435 b	35.857 ± 7.217 c	1.538 ± 0.152 ab	0.980 ± 0.018 a
	S+BR	0.035 ± 0.001 c	9.750 ± 1.350 ab	1142.220 ± 123.103 b	46.805 ± 2.875 ab	1.539 ± 0.084 ab	0.984 ± 0.006 a

**Table:** 

Days after saline-alkali stress	Treatment	AQE	Pn_max_	LSP	LCP	R_d_	R^2^ of Model fitting
		(μmol·μmol^-1^)	(μmol·m^-2^·s^-1^)	(μmol·m^-2^·s^-1^)	(μmol·m^-2^·s^-1^)	(μmol·m^-2^·s^-1^)	
49	CK	0.059 ± 0.003 a	14.098 ± 0.430 a	1409.557 ± 103.415 a	32.767 ± 2.057 b	1.792 ± 0.165 a	0.989 ± 0.005 a
	S	0.044 ± 0.006 b	5.837 ± 0.568 d	1081.930 ± 19.570 b	38.484 ± 2.034 a	1.448 ± 0.154 b	0.981 ± 0.010 a
	S+GABA	0.051 ± 0.010 ab	8.522 ± 0.367 b	1233.413 ± 136.326 b	34.390 ± 5.340 ab	1.531 ± 0.094 b	0.983 ± 0.011 a
	S+SA	0.047 ± 0.003 b	7.647 ± 0.506 bc	1198.913 ± 18.162 b	36.979 ± 1.931 ab	1.518 ± 0.059 b	0.990 ± 0.006 a
	S+BR	0.044 ± 0.006 b	6.992 ± 0.945 c	1177.633 ± 43.000 b	36.977 ± 0.556 ab	1.433 ± 0.157 b	0.981 ± 0.004 a
63	CK	0.068 ± 0.008 a	10.919 ± 0.440 a	1366.237 ± 92.838 a	28.710 ± 1.193 c	1.738 ± 0.107 a	0.994 ± 0.005 a
	S	0.026 ± 0.004 c	4.113 ± 0.666 c	1020.449 ± 28.359 c	47.165 ± 7.446 a	1.044 ± 0.115 c	0.981 ± 0.014 ab
	S+GABA	0.042 ± 0.005 b	7.186 ± 0.759 b	1190.727 ± 71.025 b	33.997 ± 3.594 bc	1.264 ± 0.163 bc	0.989 ± 0.005 ab
	S+SA	0.048 ± 0.008 b	7.213 ± 1.230 b	1247.513 ± 101.793 ab	34.380 ± 3.042 bc	1.436 ± 0.105 b	0.989 ± 0.004 ab
	S+BR	0.038 ± 0.002 b	6.094 ± 0.516 b	1101.683 ± 96.673 bc	36.664 ± 1.048 b	1.226 ± 0.054 bc	0.972 ± 0.017 b
84	CK	0.063 ± 0.007 a	9.433 ± 1.016 a	1210.533 ± 86.082 a	35.789 ± 1.322 d	1.965 ± 0.133 a	0.994 ± 0.003 a
	S	0.030 ± 0.005 c	1.482 ± 0.586 d	629.706 ± 89.028 c	65.133 ± 5.161 a	1.247 ± 0.127 c	0.977 ± 0.023 a
	S+GABA	0.039 ± 0.003 bc	6.306 ± 0.630 b	972.095 ± 61.818 b	44.791 ± 2.175 c	1.512 ± 0.074 b	0.986 ± 0.005 a
	S+SA	0.040 ± 0.003 b	6.258 ± 0.584 b	931.962 ± 36.369 b	42.439 ± 1.307 c	1.487 ± 0.038 b	0.993 ± 0.004 a
	S+BR	0.030 ± 0.005 c	3.261 ± 0.171 c	904.133 ± 91.381 b	54.687 ± 4.474 b	1.293 ± 0.063 c	0.984 ± 0.004 a

The maximum net photosynthetic rate across all treatments was around 8 mol m-2 s-1 prior to the stress treatment, but by DAA 21, it dropped to 4.841 mol m-2 s-1 in the S-treated group, which was only 46.61% of that in the control. The maximum net photosynthetic rates of S+GABA, S+SA and S+BR at this time were 6.971 μmol m^-2^ s^-1^, 7.294 μmol m^-2^ s^-1^ and 7.506 μmol m^-2^ s^-1^, respectively. This indicated that the grapevine plants sprayed with growth regulators could all alleviate salinity stress to some extent, and the BR regulator was the most effective at this time.

Apparent quantum efficiency (AQE) indicates the slope of the light response curve in the low light phase. All groups showed light absorption values around 0.04 μmol m^-2^ s^-1^ before the stress treatment. The apparent quantum efficiency of the control group gradually increased with the growth and development of the grape plants, while the S-treated group showed an overall decreasing trend with the prolongation of the stress time and was only 38.24% of the control group at 63 D. The apparent quantum efficiency of grapevine plants sprayed with growth regulators showed a decreasing and then increasing trend after stress. All three regulators significantly increased the leaf light energy conversion efficiency at 63 D after stress.

In terms of light compensation points, the S treatment group gradually increased as the stress level increased. While GABA and SA had a substantially better impact than BR. Moreover, in terms of dark respiration rate, a significant difference began to appear between the control group and the S treatment group at 35 D after stress. Saline stress reduces the dark respiration rate of plants. At 84 D of stress, the S group dropped to the lowest value of 1.247 μmol m^-2^ s^-1^, which was only 63.46% of the control group. In contrast, the dark respiration rate of treatment groups (GABA and SA) increased by 21.25% and 16.14%, respectively, as compared to the S group, which significantly improved the ability of the grape plants to tolerate stress.

### 3.5 Effect of exogenous regulators on chlorophyll fluorescence of grape leaves under salinity stress

**Figure 5:**
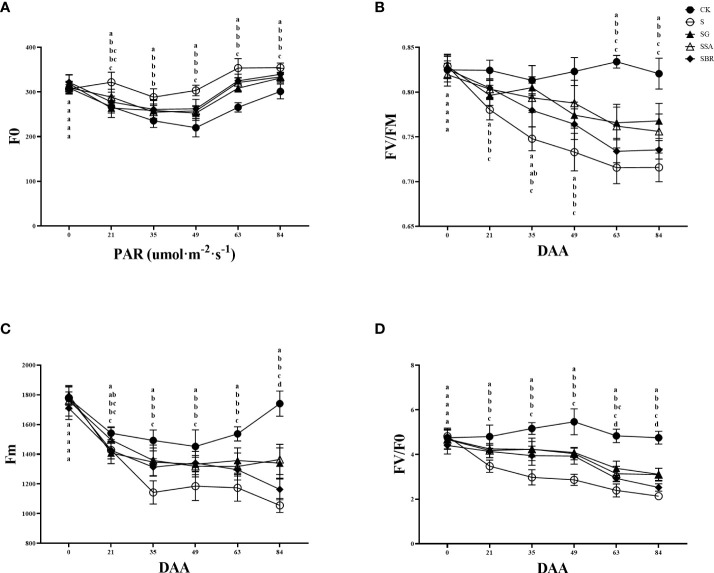
Effects of plant regulators on chlorophyll fluorescence parameters of grapevine leaves under salt alkali stress. **(A)** F0. **(B)** Fv/Fm. **(C)** Fm. **(D)** Fv/F0. Significant differences among samples have been displayed by a-e letters (one-way ANOVA, P<0.05). Error bars = ± SE (n≥3).

The changes in chlorophyll fluorescence parameters can reflect the primary reaction of photosynthesis, electron chain transfer and CO_2_ fixation process in plant leaves. Fv/Fm is the maximum photochemical quantum yield of photosystem II (PS II), which can identify the plant resistance as shown in **Figure 5**. In this study, the maximum photochemical quantum yield of PS II in the control group constantly fluctuated between 0.8 and 0.85, while the saline treatment group showed an overall decreasing trend. At 63 D of salinity stress, there was no longer a significant difference between the S+BR-treated and S-treated groups. While the Fv/Fm values of the S+GABA and S+SA-treated groups were still significantly higher than those of the S-treated group at this time (**Figure 5B**).

The initial fluorescence (F0) showed a trend of decreasing and then increasing with the prolongation of saline stress in all groups, and the S group was always higher than the other groups. At 84 D of saline stress, the treatment groups with GABA, SA and BR increased by 10.02%, 10.79% and 12.73%, respectively, all of which significantly alleviated saline stress. There was no significant difference among the three regulators in terms of improvement of F0 (**Figure 5A**).

Salinity stress causes a decrease in the maximum fluorescence (Fm) of grapevine, and at 21 D after stress treatment, it can be found that the Fm of the S group is significantly lower than that of the control group. After that, the maximum fluorescence values of the S group showed a decreasing trend. It was observed that the three regulators, GABA, SA and BR played a significant mitigating role and had similar effects (**Figure 5C**).

Fv/F0 represents the potential photochemical activity of PSII. Treatment groups consistently showed a decreasing trend under salinity stress. The effects of the three regulators (GABA, SA and BR) on Fv/F0 were similar from 21 to 63 D of stress. However, by 84 D of stress, only the S+SA and S+GABA groups were still playing a role in regulating the resistance of grapevine plants to the adverse effect of stress (**Figure 5D**).

### 3.6 Effect of exogenous regulators on the antioxidant capacity of grape leaves under saline stress

**Figure 6:**
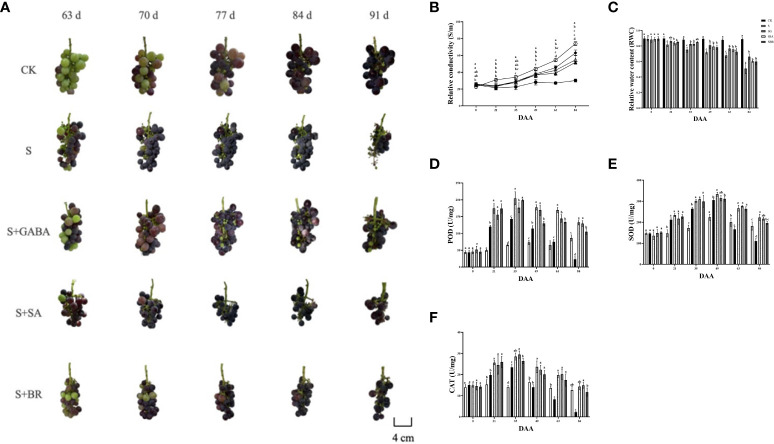
Effects of plant regulators on antioxidant enzymes of grapevine leaves under saline-alkali stress. **(A)** Berry phenotype. **(B)** Relative conductivity. **(C)** Relative water content. **(D)** POD. **(E)** SOD. **(F)** CAT. Significant differences among samples have been displayed by a-e letters (one-way ANOVA, P<0.05). Error bars = ± SE (n≥3).

When plants undergo stress conditions, cell membranes are disrupted and membrane permeability increases, causing extravasation of intracellular electrolytes and resulting in an increase in the relative conductivity of the leaves (Upadhyay et al., 2018). The relative conductivity of the control group constantly fluctuating during the stress treatment as mentioned in **Figure 6A**. Moreover, the relative conductivity of the S group increased as the severity of stress increased and reached a maximum value of 74.024% at 84 D, i.e., after harvesting, which was 2.46 times higher than that of the control group at this time. In addition, the S+GABA, S+SA, and S+BR groups all showed a decreasing and then increasing trend and reached the lowest values at 21 D, 22.913%, 23.694%, and 24.061%, respectively, which were not significantly different from the control group. It indicated that exogenous application of GABA, SA, and BR could significantly alleviate salinity stress damage in the early stage of stress. There was no significant difference among the three regulators. At 84 D after stress treatment, the conductivity of S+GABA, S+SA, and S+BR groups were 51.80%, 54.79%, and 63.02%, respectively, which were 0.70, 0.74 and 0.85 times higher than that of the S group. It showed that exogenous application of GABA and SA at the late stage of stress relieved significantly and substantially better than BR.

The relative water content of grape leaves in the S group decreased sharply with the increase in the incidence of salinity stress. In contrast, the S+GABA, S+SA, and S+BR groups showed a trend of decreasing gradually initially, as shown in **Figure 6B**. The relative water content of grape leaves in the S+GABA group was 86.28% at the 21st D of salinity stress treatment, which was significantly higher than that of 81.71% in the S group. At the same time, there was no significant difference between the S+SA, S+BR, and S groups. The relative water content of grape leaves in the S+GABA, S+SA, and S+BR treated groups was 66.27%, 60.29%, and 59.89%, respectively, which was significantly higher than that in the S group at 84th D of salinity stress treatment and increased by 30.17%, 18.42%, and 17.64% respectively compared to the S group. It indicated that exogenous applications of GABA, SA, and BR were all effective in alleviating salinity stress at the late stage of salinity stress treatment.

Antioxidant enzyme activity increases dramatically in plants under saline stress. As can be seen from **Figure 6C**, with the increasing of salinity, all treatment groups under salinity stress showed a trend of sharp increase followed by a gradual decrease and all of them reached the highest POD activity at 35 D. S, S+GABA, S+SA and S+BR groups were 142.80, 204.48, 176.64 and 198.9, respectively, which were approximately 2.20, 3.14, 2.71 and 3.05 times higher, respectively than those of the control group. At 84 D of salinity stress after harvesting, the POD activity of the S group decreased sharply to 23.28, which was only about one-fourth of that of the control group (**Figure 6D**). The POD activity of the S+GABA, S+SA, and S+BR groups was 132.72, 129.36, and 105.12, respectively, which was about 1.5, 1.5, and 1.2 times, respectively, that of the control group. It indicated that the spraying of GABA and SA is more effective in alleviating stress at the late stage of salinity stress.

The trend of SOD activity in each group was similar to that of POD. The peak of enzyme activity in each group was obtained on the 49th day of salinity stress (**Figure 6E**). At 63 D of saline stress, the SOD activity of the S group began to lower than that of the control and by 84 D, it was 110.29, which was only about 60% of that of the control group. It indicated that the resistance to severity of the S group was weakened at the later stage of stress. The S+GABA, S+SA, and S+BR groups were consistently higher than the S and CK groups. From the 49th D after salinity stress, the CAT activity of the S group started to lower than that of the CK control, and by 84 D, it was 2.25, which was only about 18% of that of the control group. The CAT activity of the S+GABA, S+SA, and S+BR groups was always significantly higher than that of the S group (**Figure 6F**). It showed that exogenous spraying of GABA, SA, and BR could significantly alleviate salinity stress, and mostly, these three treatments have similar effects.

### 3.7 Effect of exogenous regulators on fruit development and quality of grapes under salinity stress

Salinity stress induces earlier fruit ripening, as illustrated in **Figure 6A**. At 63 D of applying stress, the control treatment group started modifying color to enter the color transformation stage, while the S group was close to the final phase of color change. Compared to the S group, the spraying of GABA and BR regulators delayed the ripening of grape clusters.

**Figure 7:**
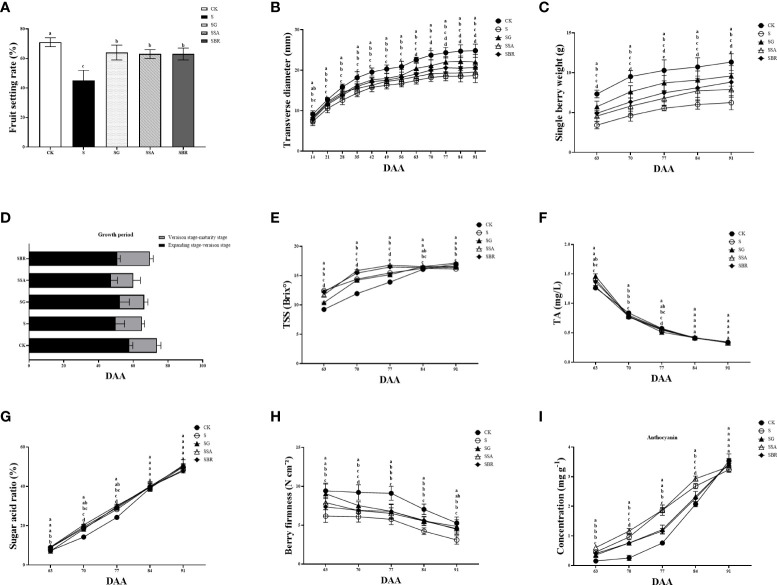
Effects of plant regulators on fruit quality of grapevine leaves under saline-alkali stress. **(A)** Fruit setting rate. **(B)** Traverse diameter of berry. **(C)** Longitudinal diameter of berry. **(D)** Growth period. **(E)** TSS. **(F)** TA. **(G)** Sugar acid rate. **(H)** Berry firmness. **(I)** Anthocyanin content. Significant differences among samples have been displayed by a-e letters (one-way ANOVA, P<0.05). Error bars = ± SE (n≥3).

The harvesting yield is significantly impacted by the fruit growth rate **Figure 7A** demonstrates that under saline stress, the fruit development rates for the S, S+GABA, S+SA, and S+BR groups were 45%, 64%, 63%, and 63%, respectively. The fruit set rate of the S+GABA group, S+SA group, and S+BR group was 42.22%, 40%, and 40%, respectively, which were significantly higher than that of the S group. It shows that exogenous applications of GABA, SA, and BR were all able to significantly increase the fruit set of grapes as these treatments almost showed similar results. **Figure 7B** reveals that although the S+GABA, S+SA, and S+BR treated groups did not differ substantially from the S group in terms of time duration of grape fruit color change at the expansion-transformation stage, all were significantly less than the control group. At the color transformation stage, the SB+R treated group took 19 D to change the color, which was 4 D longer than that of the S group. All treatment groups’ fruit cross-diameter variations clearly displayed the “double S” curve features, while the characteristics of the S treatment group under salt stress were significantly less visible, as illustrated in **Figure 7C**. Overall, the slope of fruit cross-diameter growth was steeper at 14-35 D and 56-63 D after the stress treatment when they were at the fruiting and color transformation phase. The effect of the three regulators on the improvement of grape fruit size was in order GABA > BR > SA. The fruit weight of each group showed a sharp increase and then a slow increase between 63 D and 91D after applying stress treatment, as shown in **Figure 7D**. The fruit weight of the control group was always significantly higher than that of each treatment group under saline stress. At 91 D of stress, the single fruit weight of S+GABA, S+SA, and S+BR treatment groups increased by 53.60%, 26.40%, and 41.28%, respectively, compared to the S treatment group.

At 63 D of salinity stress, the fruits of the control treatment group had just entered the color transformation phase, while the fruits of all four salinity treatment groups were in the late middle color transformation phase, as can be seen in **Figure 7E**. At this time, the soluble solids content of the S, S+GABA, S+SA, and S+BR groups was significantly higher than that of the control group. At 77 D of salinity stress, the soluble solids content of the S+SA and S+BR groups increased by 1.3% and 1.0%, respectively. In contrast, the soluble solids content of the S+GABA group decreased by 0.3% compared with that of the S group. After the 84th D of the stress treatment, the soluble solids content gradually decreased in the S group, accompanied by a slightly decreased in the S+SA and S+BR groups, but it slowly increased in the S+GABA group. The soluble solids content of the S+GABA treated group was as high as 17.13% at the 91st D of the treatment, similar to that of the control group.

The titratable acid in the fruits of all treatment groups showed a sharp decrease followed by a slow decrease from the 63D to the 91D of salinity stress, as shown in **Figure 7F**. On the 70th day of treatment, the S, S+GABA, S+SA, and S+BR groups were not substantially different and were all significantly lower than the control group. After the 84 D of treatment, i.e., the late stage of grape fruit ripening, there was no significant difference in titratable acid between all groups, which decreased to about 0.35%.

The solid-to-acid ratio is an important indicator of the flavor of the fruit. When the solid-to-acid ratio of grapes is greater than 30%, the flavor begins to be sweet. With increasing the incidence of saline stress treatment, the S, S+SA, S+GABA, and S+BR groups all showed a linear increase, while the control group showed a slow increase followed by a linear increase, as shown in **Figure 7G**. The acid fixation ratio of the S+SA group was significantly higher than that of the S group at 70-77 D of the salinity stress treatment. It indicated that SA spraying could improve the acid fixation ratio of grapevine plants under salinity stress at the color transformation phase.

As the severity of salinity stress treatment increases, the fruit hardness of each treatment group showed a trend of maintaining steady and then sharply decreasing at the stage of fruit color change to ripening, i.e., from 63 D to 91 D of stress, as can be seen from **Figure 7H**. The S, S+SA, S+GABA, and S+BR groups always had higher fruit hardness than the S group. At 91 D of stress treatment, the fruit hardness of S+GABA, S+SA, and S+BR groups was 4.86 N/cm2, 4.56 N cm^-2,^ and 4.47 N cm^-2^, respectively, which were 56.27%, 46.62% and 43.73% higher than those of S group, respectively.

From the color transformation stage to the ripening stage, the anthocyanin content in the skin starts to accumulate, resulting in a gradual change in skin color from green to purple. The total anthocyanin content of all four treatments of saline stress was higher than that of the control at 63-84 D of the treatment, i.e., from the color transformation stage to the ripening stage, revealing that saline stress induced color change in the grapevines as shown in **Figure 7I**. At 77 D of treatment, the total anthocyanin content of S+SA and S groups was significantly higher than that of S+GABA and S+BR groups. There was no significant difference between the S+SA and S groups as they both showed the same value of 1.87 mg g^-1^ while S+GABA and S+BR groups showed anthocyanin values of 1.21 mg g^-1^ and 1.16 mg g^-1^, respectively.

### 3.8 Correlation analysis between plant growth regulators and physiological parameters

**Figure 8:**
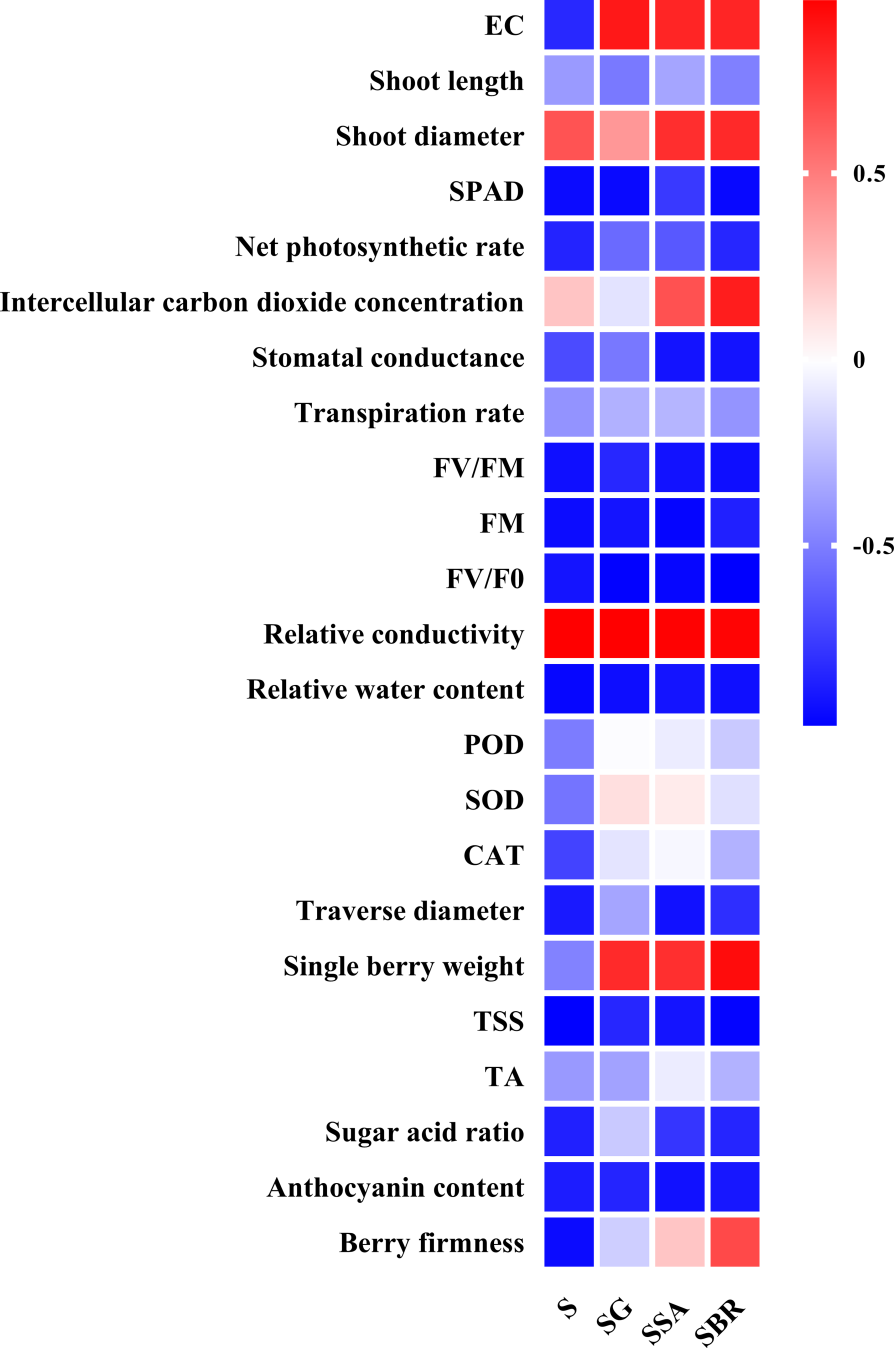
The correlation analysis among different plant growth regulators (γ-aminobutyric acid (GABA), salicylic acid (SA), and brassinolide (BR)), leaf photosynthetic index and berry quality-related parameters.

To further explore the relationship between plant growth regulators, leaf physiological indicators, and fruit quality indicators, we also conducted a correlation analysis. As shown in **Figure 8**, almost all parameters decreased under salt alkali stress (except for shoot diameter, Intercellular carbon dioxide concentration, and relative conductivity). Specifically, GABA has a promoting effect on the growth of soil EC, SOD, and single berry weight. SA has a significant promoting effect on soil EC, shoot diameter, intercellular carbon dioxide concentration, relative conductivity, single berry weight, TA, and berry firmness. In addition, BR also has a significant gain effect on soil EC, shoot diameter, intercellular carbon dioxide concentration, and single berry weight. Therefore, the following judgment was made: GABA, SA, and BR had a significant effect on restoring leaf damage and reducing berry quality under salt alkali stress. These three plant growth regulators have different gain effects on different parameters, but they all have a significant promoting effect on new shoot diameter, soil EC, and single berry weight.

## 4 Discussion

Salinity stress affects the plant phenotype (Mzid et al., 2018; Zhu et al., 2019; Lu et al., 2022). In this experiment, the effects of exogenous regulators GABA, SA, and BR on grape growth under salinity stress were investigated in terms of leaf performance, basal diameter of new shoots, new shoot length, leaf abscission, chlorophyll content, photosynthetic characteristics, light response curve, and chlorophyll fluorescence. The results showed that the exogenous application of GABA, SA, and BR on grape leaves under salinity stress could reduce the degree of leaf phenotypic damage, increase chlorophyll content, and reduce leaf abscission caused by salinity stress. GABA, SA, and BR were unable to affect the size of the base diameter of grape new shoots under salinity stress but could slightly promote new shoot elongation at the pre-and mid-growth stages. Grape leaves sprayed with exogenous GABA, SA, and BR all improved the photosynthetic capacity of the plants by increasing Pn, Gs, and Tr and decreasing Ci. Grape leaf treated with exogenous application of GABA, SA, and BR increased significant light response parameters Pnmax, AQE, and LSP and decreased LCP. Spraying of exogenous GABA, SA, and BR on grape leaves significantly increased Fm and Fv/Fm but decreased fluorescent chlorophyll parameters such as F0 and Fv/F0. In conclusion, the exogenous regulators GABA, SA, and BR had a mitigating effect on the growth of grapes under saline conditions. Overall, GABA and SA have the same mitigating effects. Still, BR has less mitigating effect as compared to both of these, as the mitigating effect of BR diminished in the later stages of saline stress.

Under normal conditions, biofilms are selectively permeable and play a key role in ensuring the stability of the intracellular environment. However, saline stress decreases the function of the cytoplasmic membrane and electrolyte extravasation, which in turn affects the photosynthesis, physiological metabolism, and other growth and development processes of plants and eventually causes different degrees of salt damage to the plants (Zhao et al., 2021). GABA, SA, and BR are important plant life activity regulators as they can resist the accumulation of toxic substances by regulating the antioxidant enzyme activity in plants (Li et al., 2021; Zhao et al., 2021; Yang et al., 2023). In this experiment, the effects of exogenous regulators GABA, SA, and BR on the antioxidant activity of grape leaves under saline stress were also investigated. The relative conductivity, relative water content, and antioxidant enzyme activity were measured, respectively. Overall, the results showed that the spraying of exogenous GABA, SA, and BR on grape leaves under salinity stress reduced the relative conductivity of leaves, increased the relative water content of leaves as well as increased the activities of POD, SOD, and CAT antioxidant enzymes. Finally, It reflects that GABA and SA have the same mitigating effects, but BR has less alleviating effects as compared to both because of the protective ability of BR on cytoplasmic membrane permeability and the promotion of enzyme activity used as a gradual weakening in the late stage of stress.

The commercial value of the fruit yield is the most important goal for agricultural producers, and the color of the berry is the most intuitive indicator to determine the ripening phase. Previous studies have shown that salinity stress stimulates fruit coloration along with the accumulation of anthocyanins and advances ripening in “Kyoho” grapes (Li et al., 2013). However, the application of exogenous growth regulators to improve grape fruit quality needs to be further explored. Therefore, the effects of exogenous regulators GABA, SA, and BR on grape fruit growth and quality under salinity stress were also investigated in this experiment. Relative fruit set rate, color change rate, fruit cross-sectional diameter, fruit weight per fruit, soluble solids, titratable acid, solid to acid ratio, fruit hardness, and total fruit anthocyanin content were determined, respectively. The results showed that exogenous GABA, SA, and BR sprayed on grape leaves under salinity stress could improve fruit sets. And there was no significant difference between the effects of these three regulators. Plants treated with exogenous application of GABA, SA, and BR improved fruit cross diameter, fruit weight, and hardness under salinity stress, with GABA having the most significant effect against salinity stress. Foliar sprays of SA had a marginally favorable effect on accelerating the time from expansion of the fruit set to color transformation under salt stress, whereas BR significantly prolonged the period from color transformation to the ripening stage. Salinity stress accelerates fruit ripening, accompanied by an increase in soluble solids and a decrease in titratable acids. At 63 to 77 D of stress, SA and BR could accelerate the accumulation of soluble substances in fruits. GABA, SA, and BR could accelerate the degradation of titratable acids in fruits to improve the fruit solid-acid ratio. However, there were no significant differences among treatments at the late fruit ripening stage, i.e., at 84 and 91 D of stress. Salinity stress increased the accumulation of anthocyanin in the pericarp. From 63 D to 84 D of stress, leaf spraying with exogenous SA further promoted anthocyanin accumulation; in contrast, GABA and BR reduced anthocyanin’s early ripening stages to improve content.

## 5 Conclusion

In conclusion, this study investigated the effects of exogenous sprays of GABA, SA, and BR on the growth and development of grapes under salinity stress by means of foliar sprays. The studies were conducted on grape growth, leaf antioxidant activity, and fruit development to provide strong data to support that exogenous spraying of GABA, SA, and BR on grape leaves under saline stress can alleviate saline damage and promote the growth and development of fruit trees. The main conclusions are as follows: the exogenous regulators GABA, SA, and BR all alleviated the inhibition of grape growth by saline stress. Exogenous application of GABA, SA, and BR alleviated leaf abscission produced by salinity stress and promoted the growth of new shoots as well as the accumulation of chlorophyll content. In addition, the exogenous application of regulators GABA, SA, and BR alleviate the damage to the antioxidant mechanism of grape leaves by salinity stress as well as can regulate the effect of salinity stress on grape fruit development and quality. In terms of external fruit quality, saline stress stimulates fruit color change and ripening. SA treatment speeds up fruit ripening, while BR treatment, on the contrary, relatively prolongs the period from fruit color transformation to ripening. In addition, GABA, SA, and BR significantly increase fruit set, fruit diameter, fruit weight per unit, and anthocyanin content. In terms of intrinsic fruit quality, the spraying of GABA, SA, and BR on leaves increased the content of soluble solids. It also reduced the content of titratable acid and increased the solid to acid ratio during the color transformation and early ripening stages to improve the flavor quality of the fruit in stress-treated plants.

## Data availability statement

The original contributions presented in the study are included in the article/supplementary material. Further inquiries can be directed to the corresponding authors.

## Author contributions

MZ: Data curation, Formal Analysis, Writing – review & editing. JL: Formal Analysis, Writing – original draft. XS: Writing – review & editing. MS: Writing – review & editing. YQ: Writing – review & editing. DG: Writing – review & editing. LW: Funding acquisition, Investigation, Supervision, Validation, Writing – review & editing. SW: Funding acquisition, Investigation, Supervision, Validation, Project administration, Writing – review & editing.
